# Transcriptome analysis provides insights into the regulatory function of alternative splicing in antiviral immunity in grass carp (*Ctenopharyngodon idella*)

**DOI:** 10.1038/srep12946

**Published:** 2015-08-07

**Authors:** Quanyuan Wan, Jianguo Su

**Affiliations:** 1 College of Fisheries, Huazhong Agricultural University, Wuhan 430070, China; 2 Freshwater Aquaculture Collaborative Innovation Center of Hubei Province, Wuhan 430070, China

## Abstract

Characterization of the transcriptomic response to infection is an effective approach to understanding the immune mechanisms. Herein we challenged grass carp (*Ctenopharyngodon idella*) with grass carp reovirus (GCRV) and sequenced four cDNA libraries obtained from head-kidney and spleen by using Illumina Miseq. As a result, we gained a total of 21.52 Gb clean data with 107.96 million reads, and *de novo* assembled 55,199 unigenes with an average length of 1,470 bp. Comparative transcriptome analysis reveals that 217 unigenes are differentially expressed (fold-change of at least 4) between resistant and susceptible fish in both head-kidney and spleen, and of which 36 unigenes were validated by RT-qPCR experiment. The expression profile of immune-related genes demonstrates that the immune response of spleen is more intense than that of head-kidney. Remarkably, 11,811 unigenes contain multiple transcripts, of which 322 unigenes possess notably differentially expressed transcripts between the four transcriptomic datasets. Furthermore, the splicing transcripts of *IL-12p40* and *IL-1R1* are firstly found to play diverse roles in the antiviral response of fishes. This study provides a complete transcriptome dataset of *C. idella*, which is valuable for the studies of immune complexity and, moreover, throws light on the regulatory role of AS in antiviral immunity.

Alternative splicing (AS) of mRNA precursors as a common mechanism regulating gene expression can increase the diversity of the transcriptome and expand the information content^1^. In many cases, each mRNA transcript encodes a unique polypeptide with a certain biological function. In other cases, some of the mRNA transcripts might generate aberrant splicing events with little or no functional consequence, but rather function as an ancillary pathway coupled to nonsense-mediated mRNA decay (NMD)^2^,^3^. As such, AS can control cellular function to regulate development, physiology, and response to stimuli. In recent years, with the development of high-throughput sequencing technologies, although much attention were paid to the genome-wide analysis of AS^4^,^5^, much less researches were focused on the AS regulation in the immune systems. In practice, a few genes (listed in the review literatures^1^,^6^) involved in the regulation of human T cell responses to antigen are identified to be AS regulated, which broadens the perception of immune systems. Due to fish is one of the lower vertebrates, AS studies on fishes should be widely developed, which might contribute to the understanding of fish genome and immune systems evolution^7^,^8^. As important economically animals, fishes should be investigated for AS regulation in the immune system as well, which might contribute to the disease control in aquaculture. In this context, AS of Japanese flounder (*Paralichthys olivaceus*) has been recently detected by using RNA sequencing^9^, which lays a foundation for functional genomics studies in fishes.

Grass carp (*Ctenopharyngodon idella*), belonging to the family *Cyprinidae*, is an economic fish of the highest yield in China and even in the world, but it seriously suffers from hemorrhagic disease caused by grass carp reovirus (GCRV). In recent years, lots of prevention treatments were implemented but little effect^10^. At present, 20,215 expressed sequence tags (ESTs) of *C. idella* are publicly available in NCBI database, of which only about 5,300 ESTs are related to immune pathways. And heretofore, merely a small number of immune-related genes are completely cloned and characterized. Although previous analyses of ESTs^11^, cDNA tag library^12^ and even expression profiles^13^,^14^ contributed to the understanding of *C. idella* immune responses, the biologic information for the global immune analysis of *C. idella* may still be scarce. Therefore, more complete and unbiased transcriptome datasets should be established for the identification of novel genes, isoforms, AS events and genetic markers.

In teleost fish, head-kidney with the highest concentration of developing B lymphoid cells is an important organ involved in adaptive immunity, and is also the major source of antibody production^15^. Similarly, spleen as another immune organ involved in innate immunity plays vital roles in haematopoiesis, antigen trapping and degradation, and antibody production processing^15^. Given this, the RNA-seq of head-kidney and spleen is of great significance for revealing the immune system of teleost fish. Herein, the transcriptome libraries of the head-kidney and spleen samples from GCRV-challenged *C. idella* were constructed by using MiSeq platform which can yield highest quality reads with lowest error rate^16^,^17^. These libraries were aimed at: 1) creating a high-quality unigene library as a database for gene annotation, differentially expressed gene (DEG) analysis, novel gene discovery, and AS events identification, which will benefit future researches on genome, transcriptome, and proteome; 2) characterizing the difference of immune response between resistant and susceptible fish, and the various reactions between head-kidney and spleen during GCRV infection. Furthermore, AS events of *IL-12p40* gene, *IL-1 receptor type I* gene (*IL-1R1*) and *Immune-related lectin-like receptor* genes (*ILLRs*) were validated by using PCR amplification and rapid-amplification of cDNA ends (RACE) technology, and the expression levels were confirmed by reverse transcription quantitative real-time PCR (RT-qPCR).

## Results and Discussion

### *De novo* assembly and annotation of *C. idella* transcriptome

By Illumina MiSeq 2 × 250 bp pair-end sequence technology, a total of 107,959,648 clean reads (21.52 Gb of data bulk) with an average length of 199 bp were generated from the 4 libraries (SS1, SR2, KS3, and KR4) (Table 1). This data is approximately 15-fold larger than the genome size of zebrafish (*Danio rerio*)^18^ and 144-fold larger than the assembled transcriptome size of *C. idella*. The high-quality reads were available in the NCBI SRA browser (BioProject accession number: SRP049081). To create a high-quality unigene library, all sequencing reads from 4 libraries were together assembled. The *de novo* assembly yielded a total of 101,812 unisequences (149.64 Mb), integrated into 55,199 unigenes which is coincident with the number of genes in common carp (*Cyprinus carpio*) (52,610 protein-coding genes)^19^ and *D. rerio* (53,734 transcripts)^18^. The average size and N50 size of unigenes were 1,470 bp and 2,350 bp, respectively (Table 1 and Supplementary Fig. S1).

A feasible strategy to assess whether transcripts have actually and correctly assembled is internal validation, *i.e*., mapping clean reads back to the assembly dataset by using Bowtie software^20^. The result of validation showed that more than 83% clean reads of each library were mapped onto the assembled transcriptome sequences (Supplementary Table S1). This result indicated that the *de novo* assembly was satisfactory, and the assembled dataset could be employed for subsequent analysis. For the purpose of functional annotation, the prediction of ORFs of all the unisequences was performed by using Trinity software. The result showed that ORFs of 54,609 unisequences (53.6%) were successfully predicted, suggesting that majority of unisequences were derived from intact protein-coding transcripts. With the ORF prediction result, all assembled sequences were aligned to various databases for their functional annotation. Supplementary Table S2 shows the statistical data of functional annotation, which reveals that a total of 73,828 unisequences (72.5%) and 34,567 (66.2%) unigenes return a valid BLAST result. Supplementary Figure S2 display the BLAST top-hit species distribution of unisequence annotation, which reveals that the assembled unisequences show the highest homology to zabrafish (*D. rerio*), followed by common carp (*C. carpio*), and Nile tilapia (*Oreochromis niloticus*).

**Figure 1:**
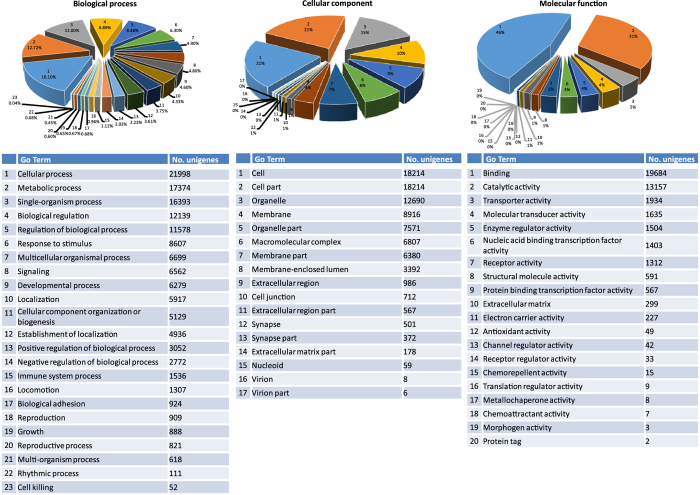
Level 2 GO term assignment to the *de novo* assembled dataset of *C. idella* and distribution in categories of biological process, cellular component and molecular function. The number on each pie shows the corresponding GO term and its percentage.

By gene ontology (GO) analysis, 32,519 of 101,812 unisequences were assigned to 8,856 GO terms (132,710 term occurrences in total) consisting of 60 GO sub-categories under three major categories (Fig. 1 and Supplementary Data 1). The annotation of the 101,812 unisequences against orthologous groups of proteins database (COG) databases yielded 14,188 putative proteins (Supplementary Table S2), of which 518 proteins were related to defense mechanisms. Overall, the COG/KOG/NOG-annotated putative proteins fell into 25 classifications, including “information storage and processing”, “cellular processes and signaling”, and “metabolism”, in accordance with the categories observed in GO annotation (Supplementary Fig. S3 and Supplementary Data 1). In addition, a total number of 27,367 unisequences (11,602 unigenes) were significantly categorized into 317 pathways, of which metabolic pathways (3,350 unisequences), pathways in cancer (1,273 unisequences), HTLV-I infection (1,072 unisequences) and PI3K-Akt signaling pathway (963 unisequences) were the four pathways containing most unisequences (Supplementary Data 1). The annotation information may be beneficial to researches on *C. idella*, even on other non-model fishes.

### Global changes in gene expression upon GCRV infection

One of the available approaches to characterizing the immune response of *C. idella* to GCRV infection is gene screening from those DEGs after a challenge experiment. To this end, global fold changes in gene expression upon GCRV infection were detected after the normalization of fragment counts of each assembled sequence. The analysis result was presented in Supplementary Data 2. Comparing resistant samples (KR4 and SR2) with susceptible samples (KS3 and SS1), we gained a total of 1,025 DEGs in head-kidney (296 up-regulated and 729 down-regulated) and 871 DEGs in spleen (476 up-regulated and 395 down-regulated) (*P* < 0.05, FDR < 0.05) (Supplementary Fig. S4a). Among these DEGs, 59 DEGs were up-regulated and 158 DEGs were down-regulated in both head-kidney and spleen (Supplementary Fig. S4b,c and Supplementary Data 3).

Furthermore, to identify significant GO categories and Kyoto encyclopedia of genes and genomes pathway database (KEGG) pathways in head-kidney and spleen of *C. idella* upon GCRV infection, GO and KEGG enrichment analyses were performed. In the analyses, the ratio of differentially expressed unisequence (DES) to the total unisequences of corresponding GO category or KEGG pathway was regarded as the major criteria for enrichment assessment. It was supposed that GO subcategories or KEGG pathways with high-ratio DESes might be the major concerns. For the sake of simplicity, the enrichment results were illustrated with Supplementary Figure S5, and the details were in Supplementary Data 4. The result reveals that GO sub-category with the highest DES enrichment ratio is “regulation of antigen processing and presentation” in head-kidney, while it is “toxin metabolic process” in spleen, suggesting head-kidney is a vital organ for antigen processing and presentation, while spleen is an important organ for toxin metabolic processing. Additionally, to single out some significant DEGs, the analysis of KEGG pathways among all the DEGs was conducted. As a consequence, *c-Fos* gene and IκBα-like protein A coding gene (*IκBαLA*) were found to be respectively up- or down-regulated in mitogen-activated protein kinase (MAPK), Toll-like receptor (TLR), RIG-I-like receptor (RLR), T cell receptor (TCR), B cell receptor (BCR), and nuclear factor-κB (NF-κB) signal pathways. Since c-Fos is a component of transcriptional factor AP-1^21^, the relatively high expression of *c-Fos* in resistant samples may induce the activity of AP-1 to regulate the expression of immune-related genes. Alternatively, the highly expressed c-Fos protein directly activates the synthesis of lipid to participate in immune responses^22^,^23^. As for *IκBαLA*, its relatively low expression may conduce to the high expression of NF-κB. As such, c-Fos and IκBαLA may play an important role in the mediation of immune response; thereby these two genes should be further investigated. With the assistance of signal network depiction from STRING database, the interactive molecules of c-Fos and IκBαLA appeared in the sight (Supplementary Fig. S6). The following gene expression analysis based on c-Fos network revealed that only the expression trend of *Jun D proto-oncogene* (*JunD*) was uniform with that of *c-Fos*. This result may indicate that JunD, but not c-Jun was the preferential molecule that interacts with c-Fos to form AP-1 in *C. idella*.

On the other hand, to investigate the difference in immune responses between head-kidney and spleen of *C. idella* during GCRV infection, gene expression levels in head-kidney and spleen were compared. A total of 4,341 and 3,908 unigenes were specifically expressed in head-kidney and spleen, respectively. In resistant samples, 2,277 unigenes were differentially expressed between head-kidney and spleen; while in susceptible samples, 155 genes were differentially expressed between head-kidney and spleen (Supplementary Data 2). Since the susceptible fish died while resistant fish survived in the challenge experiment, the antiviral response in the susceptible fish was at the early phase, while that in the resistant fish was at the later phase. Whereupon, these results testify that the head-kidney and the spleen function equally in the early stage of antiviral response, and then they function dissimilarly in the later period. This inference coincides with previous findings that head-kidney and spleen show specific sets of DEGs in response to pathogens^24^,^25^.

### Validation of RNA-seq data by RT-qPCR

**Figure 2:**
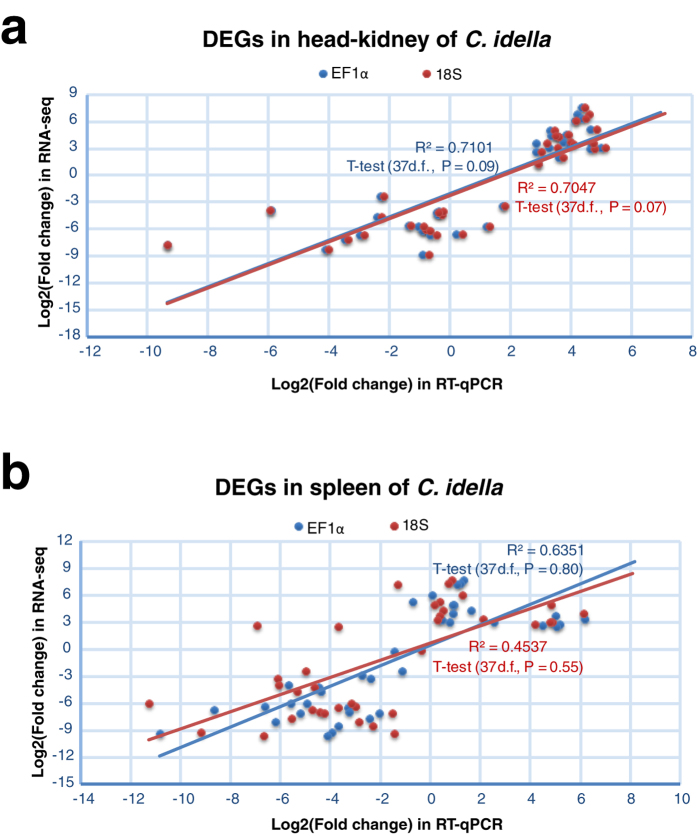
Comparison results between RT-qPCR and RNA-seq data. The expression levels were normalized to *18S rRNA* (red) and *EF1α* (blue), respectively. X axis and Y axis are the fold changes of gene expression, comparing KR4 with KS3 in section a; SR2 with SS1 in section b.

To validate the gene expression result of the RNA-seq data, 36 significant DEGs (*P* < 0.05, FDR < 0.05, fold-change of at least 8), *recombination activating gene 1* (*RAG1*), and *RAG2* were selected for RT-qPCR analysis. With *18S rRNA* and *EF1α* as reference genes, the expression levels of these 38 genes were normalized. As was anticipated, the RT-qPCR data showed a positive linear relationship with RNA-seq data (Fig. 2 and Supplementary Table S3). And there was no statistically significant difference between the two data sets (t-test *P* > 0.05). This result suggested that the RNA-seq was a positive reference for expression profiling study on the whole, and that the assembly quality of the sequences was desirable.

### Innate and adaptive immunity-related genes were differentially expressed

**Figure 3:**
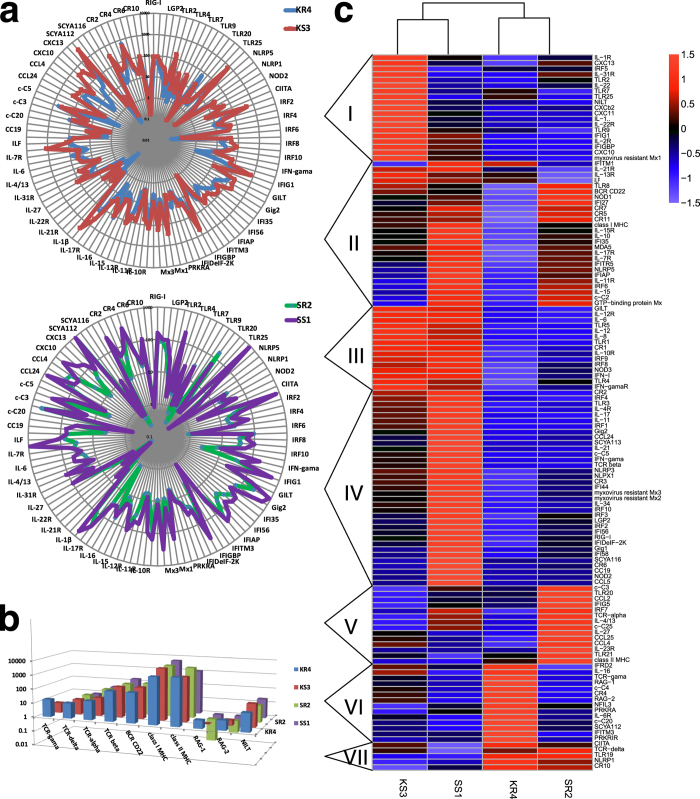
Changes in expression of immune-related genes upon GCRV infection. (**a**) Expression comparison of innate immune-related genes between susceptible and resistant libraries in head-kidney and spleen. The periphery of the circle is the X axis which presents genes; while the radius of the circle is the Y axis which presents the FPKM value of corresponding gene. The closer from the edge of the circle, the higher the expression of corresponding gene is. In this case, mostly innate immunity-related genes are relatively lowly expressed in resistant libraries. (**b**) Expression comparison of adaptive immune-related genes in head-kidney and spleen. Mostly genes are slightly higher-expressed in resistant libraries, comparing to susceptible libraries. (**c**) Two-dimensional hierarchical clustering performed on the clusters of immune-related genes which can be classified into 7 clusters named I–VII.

To characterize the immune response of *C. idella* upon GCRV infection, the expression changes of immune-related genes were observed. To this end, according to immune defense molecules listed in the literature review^26^, 128 immune-related genes were artificially chosen for gene expression analysis, and then the hierarchical cluster analysis for them was performed. The result showed that the average expression level of these innate immunity-related genes in spleen was higher than that in head-kidney, while the expression levels of these adaptive immunity-related genes in spleen were equal to that in head-kidney (Fig. 3 and Supplementary Data 5), reflecting that the immune response of spleen is more intense than that of head-kidney. In the heat map, KS3 and SS1 libraries went into one cluster, while KR4 and SR2 libraries fell into another cluster (Fig. 3c), indicating that expression changes of these 128 immune-related genes were less correlated with tissues during GCRV infection on the whole. According to the expression pattern, these 128 genes were allocated into seven clusters representing seven different expression patterns. It should be noted that the genes in the cluster VII were relatively highly expressed in KR4 and SR2. It was especially true with *TLR19*, *NLRP1* (*NACHT, LRR and PYD domains-containing protein 1*) and *CR10* (*CC chemokine receptor 10*), which might imply that they were vital molecules in antiviral responses. *TLR19* which was difficult to detect by RT-qPCR and was deduced as a tissue-specifically expressed gene in *D. rerio*^27^ is indeed rarely expressed in head-kidney and spleen of *C. idella*. In addition, since the functions of *TLR19* and *TLR20* in the non-mammalian animals have not been widely studied, to explore their specific mechanisms will be interesting.

### AS events were ubiquitous in head-kidney and spleen transcriptomes of *C. idella*

**Figure 4:**
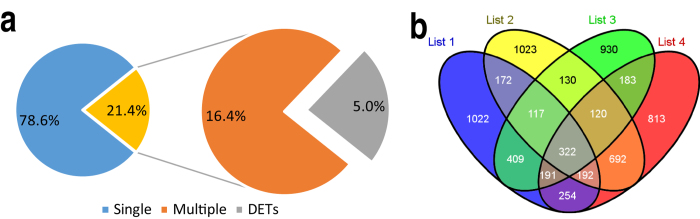
Graphical representation of the proportion of multi-transcripts genes and numbers of their differently expressed transcripts. (**a**) Apportionment of multi-transcripts containing unigenes. "Single" presents single transcript containing unigenes; "Multiple" presents multi-transcripts containing unigenes; while "DETs" stands for the multi-transcripts containing unigenes whose transcripts are differently expressed in different libraries. (**b**) Venn diagram that describes overlaps among differently regulated transcripts upon GCRV infection. List 1 and 2 respectively contain the up-regulated and down-regulated transcripts in KR4, comparing to KS3; and list 3 and 4 respectively contain the up-regulated and down-regulated transcripts in SR2, comparing to SS1. All the transcripts in the same list and from the same genes were together counted. Those overlapped genes are DETs, whose number is 2782.

The alignment between unisequences and unigenes in the present study revealed that 11,811 unigenes (21.4%) which contained multiple transcripts (at least 2 transcripts and at most 89 transcripts) may be AS regulated (Supplementary Data 6). It was worth mentioning that some transcripts of a certain gene were differentially expressed in different libraries. These genes were called differentially-expressed-transcript-containing genes (DETs). And the number of DETs was 2,782 (5% of the total unigene) (Fig. 4). Of those DETs, 322 DETs including 69 novel genes contained both up-regulated and down-regulated spliced transcripts in the head-kidney library and spleen library (Fig. 4 and Supplementary Data 6). These DETs involved in immunity, nucleotide binding, chemical modification, pre-mRNA-splicing, and other biological process. For these reasons, it is interesting to select some essential genes among these 322 DETs for further validation and exploration.

### AS events validation of the representative genes further defined the reliability of assembly data

**Figure 5:**
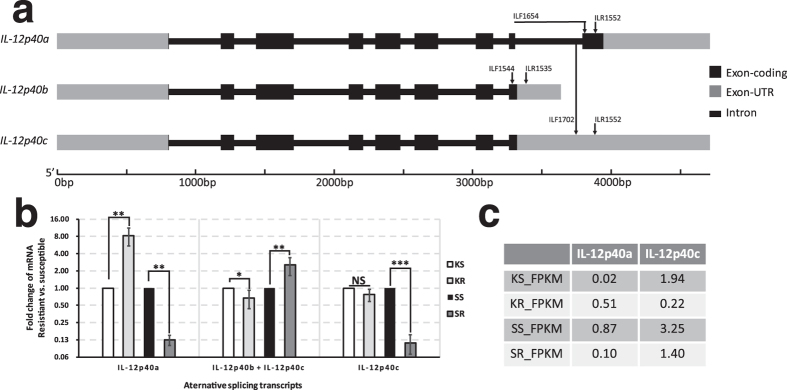
Splicing transcripts of *IL-12p40* gene and their expressions in the head-kidney and spleen of *C. idella*. (**a**) Illustration of gene structures of the splice variants. Primers for RT-qPCR validation of the gene expression are showed on the corresponding structures. Primer ILF1654 is designed at the junction of Exon 8 and Exon 9; (**b**) Fold changes of the splice variants in KR (comparing to KS) and SR (comparing to SS), which are tested by RT-qPCR. *t-test *P* value < 0.05 ** t-test *P* value < 0.01; ***t-test *P* < 0.001; ‘NS’, not significant, t-test *P* > 0.05. (**c**) The FPKM values of *IL-12p40a* and *IL-12p40c* in the RNA-seq dataset.

**Figure 6:**
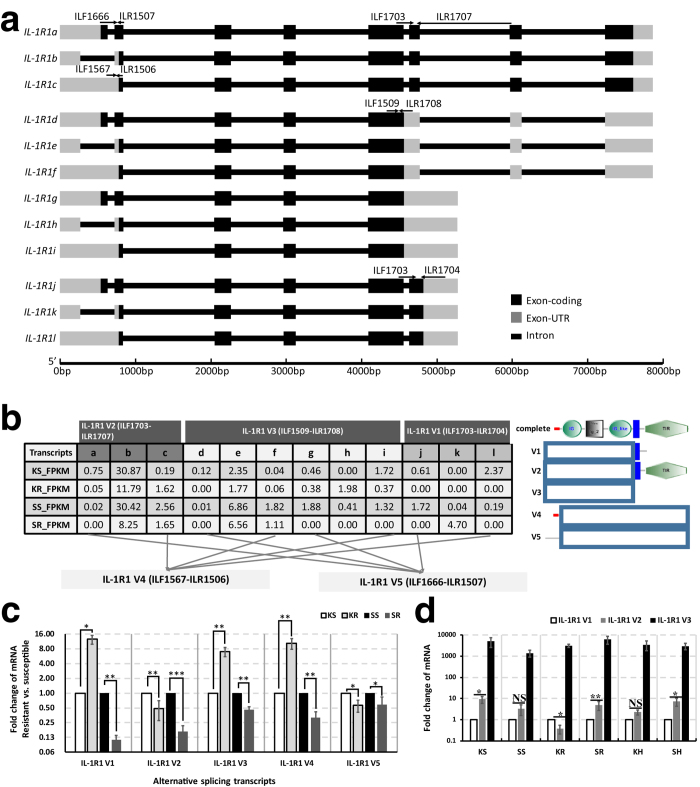
Splicing transcripts of *IL-1R1* gene and their expressions in the head-kidney and spleen of *C. idella*. (**a**) Illustration of gene structures of the splice variants. Primers for RT-qPCR validation of the gene expression are showed on the corresponding structures. Primer ILF1666, ILF1703 and ILR1707 were designed at the corresponding junctions; (**b**) The FPKM values of the 12 splice transcripts in RNA-seq dataset, and the schematic diagram of suppositional proteins ‘encoded’ by V1, V2, V3, V4 and V5 are displayed. The red parts are signal peptides, orderly followed by three Ig domains, TM domains and TIR domains. Dotted boxes are the unconcerned portion of corresponding proteins; (**c**) Fold changes of the splice variants in KR (comparing to KS) and SR (comparing to SS), tested by RT-qPCR. (**d**) Relative expression of V1, V2 and V3 in different samples. *t-test *P* value < 0.05; **t-test *P* value < 0.01; ***t-test *P* < 0.001; ‘NS’, not significant, t-test *P* > 0.05.

**Figure 7:**
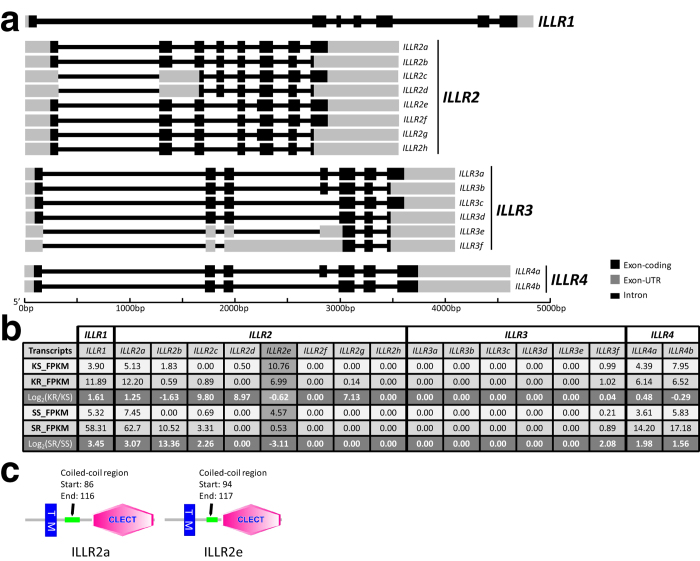
Splicing transcripts of *ILLR* genes. (**a**) Illustration of gene structures of the splice variants of *ILLR1*, *ILLR2*, *ILLR3*, and *ILLR4*; (**b**) The FPKM values of the 12 splice transcripts in RNA-seq dataset; (**c**) Predicted protein domains of ILLR2a and ILLR2e by using SMART (Simple Modular Architecture Research Tool) (http://smart.embl.de/). All protein images generated by SMART are licensed under a Creative Commons Attribution-Share Alike 3.0 Unported license. The license terms can be found on the following link: https://creativecommons.org/licenses/by-sa/3.0/

**Figure 8:**
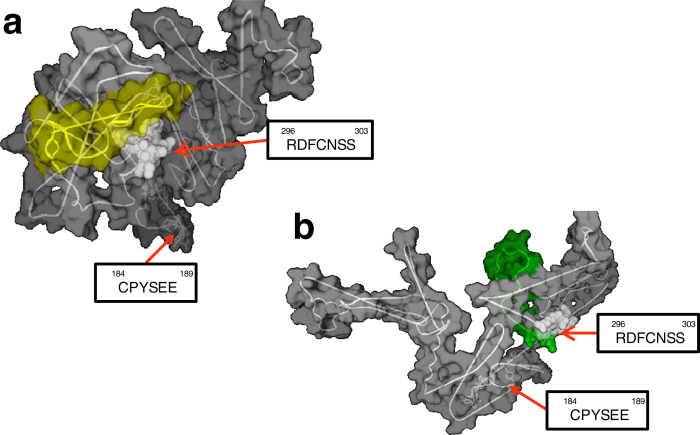
Predicted structures of IL-12p40α and IL-12p40β. According to the previous reports (reference 33), arrows and boxes show residues that are critical or important for IL-12p70 formation. (**a**) Structure of IL-12p40α. The yellow region is the additional 45 residues. It is clear that the important residues ‘RDFCNSS’ are wrapped by the carbon terminal residues, which may lead to IL-12p40α cannot dimerize with IL-12p35, thereby prevents the IL-12p70 formation. (**b**) Structure of IL-12p40β. Green region is the carbon terminal. The important residues ‘RDFCNSS’ are exposed.

In order to validate the AS events, certain representative genes were selected from 322 DETs mentioned above. *IL-12p40* gene and *IL-1R1* gene were picked out due to their coordinating function in innate and adaptive immunity^28^,^29^ and also, *ILLRs* were selected for their differential expression in the myeloid and lymphoid lineages of *D. rerio*^30^. In case of the sequencing and assembling errors, those assembly sequences should be validated by Sanger sequencing. Hence, 3′-, 5′-terminal and genomic DNA regions of these representative genes were cloned and sequenced. The comparison between the genomic DNA regions and the unisequences of these genes revealed that the *IL-12p40* gene possessed 3 splicing transcripts (Fig. 5a), that the *IL-1R1* gene contained 12 splicing transcripts (Fig. 6a), and that the *ILLR1*, *ILLR2*, *ILLR3*, *ILLR4* respectively possessed 1, 8, 6, and 2 splicing transcripts (Fig. 7a). *IL-12p40a* encodes one kind of novel p40 (375 aa, IL-12p40α), while *IL-12p40b* and *IL-12p40c* encode another one (330 aa, IL-12p40β). Furthermore, the protein structures of IL-12p40α and IL-12p40β were predicted by using SCRATCH protein predictor (free trial version, http://scratch.proteomics.ics.uci.edu/index.html) (Fig. 8). As for these representative genes, approximately 30.8% of AS events were alternative donor, 7.7% were alternative acceptor; and 12.8% were exon skipping, while 48.7% were intron retention. According to the AS validation results, it can be deduced that over 20% of *C. idella* genes are alternatively spliced during GCRV infection, which is approximate to the AS event ratio in zebrafish (*D. rerio*) (17.0%), but lower than the AS event ratios in medaka (*Oryzias latipes*) (31.2%), stickleback fish (*Gasterosteus aculeatus*) (32.4%), Japanese puffer fish (*Takifugu rubripes*) (43.2%), and human (*Homo sapiens*) (94%)^7^,^31^.

### Splicing transcripts of *IL-12p40* and *IL-1R1* were differentially expressed in the antiviral response

Firstly, the heterodimeric proinflammatory cytokines IL-12 and IL-23 are broadly documented as the major regulators of innate and adaptive immunity to induce the production of IFN-γ and facilitate T cell differentiation^28^,^32^. Three spliced transcripts of *IL-12p40* gene are found and validated, which is different from *IL-12p40* gene of *C. carpio* that three distinct transcripts encode three different proteins^33^. The FPKM values of the RNA-seq dataset indicated that these splice transcripts of *IL-12p40, IL-1R1* and *ILLR*s were differentially expressed in the four libraries (Figs 5c, 6b and 7b). Subsequently, these findings were partially validated by RT-qPCR (Figs 5b and 6c). The expression levels of *IL-12p40a* and *IL-12p40c* in KR were respectively 8.3-fold higher and 1.3-fold lower than those in KS (25.5-fold higher and 8.8-fold lower in RNA-seq data, respectively); and in SR, they were respectively 8.3- and 11.1-fold lower than those in SS (8.7- and 2.3-fold lower in RNA-seq data, respectively) (Fig. 5b,c). In addition, the result of RT-qPCR using primer pair ILF1544 and ILR1535 presented the summational expression level of *IL-12p40b* (GenBank accession no. KF944668) and *IL-12p40c*. This summational expression level was down-regulated (0.67-fold) in KR compared with that in KS, and up-regulated (2.55-fold) in SR compared with that in SS. These results were similar to the RNA-seq data that the expression levels of *IL-12p40a* were higher in KR and SS than those in KS and SR, respectively. On the contrary, the expression of *IL-12p40b* and *IL-12p40c* were lower in KR and SS than those in KS and SR, respectively (Fig. 5). Based on these findings, a hypothesis was proposed that IL-12p40α and IL-12p40β can polymerize with different monomers so as to play adverse roles in immune responses. Actually, this hypothesis is well-founded, considering the following points: 1) the chain sharing is a key feature of the IL-12 family of cytokines but the rationale for chain preference is unclear^34^; 2) there is a debate on whether IL-12p40 is positive or negative to T cell^35^,^36^,^37^; 3) IL-12p40 is able to generate multiple novel composites in combination with other locally available polypeptide partners after secretion^38^. In the case of *C. idella* IL-12p40, IL-12p40α is 45 aa longer in the C-terminal than IL-12p40β, and this difference lead to the diverse protein configurations (Fig. 8). It is hypothetical that the configuration of the novel IL-12p40α is aberrant, because that its key residues involved in dimerization are unexposed, which lead to the obstruction of the formation of IL-12p70; thereby IL-12p40α may be homodimerized to shape IL-12p80, then to induce the DC migration and T cell priming^35^,^37^. However, the redundant IL-12p40β may be polymerized with other polypeptide partners for instance IgG2b to play a negative regulatory role in immune responses^36^. This hypothesis concerning AS may provide a rational explanation for the two-sided functions of IL-12p40 in immune responses, and may imply the critical role of IL-12p80 in the later stage of antiviral responses. In view of this, the expression patterns of IL-12*p40a* and *IL-12p40b* + *IL-12p40c* are aimed at activating T cells and inducing DC migration in head-kidney, but suppressing the immune responses to recover the normalcy in spleen (Fig. 5). In terms of the expression pattern of *C. idella IL-12p40c*, which possesses a long 3′-UTR, its relative low expression in SR may be involved in the microRNA-targeted degradation^39^, aiming at preventing over-expression of the splicing mRNA-encoded protein^1^. From this view, the AS event of *IL-12p40* gene plays an important regulatory role in the antiviral responses of *C. idella*.

Secondly, because *IL-1R1* held 12 splicing transcripts, their expression levels should be separately validated. However, those splice transcripts were highly similar to each other, so it was hard to design the primers for RT-qPCR experiments aimed at differentiating the expression level of each transcripts. Considering that, those transcripts were divided into two categories by the organization of IL-1R1 protein. One category focused on the C-terminal in three forms: transmembrane (TM) (IL-1R1 V1), Toll/Interleukin receptor (TIR) and TM (IL-1R1 V2), and absence of TIR or TM (IL-1R1 V3). Another category focused on the N-terminal in two forms: signal peptide (IL-1R1 V4) and absence of signal peptide (IL-1R1 V5) (Fig. 6). Thus, by this classification, the expression levels of IL-1R1 transcripts were validated by using five pairs of primers in RT-qPCR experiment. The comparison of gene expression levels between KR and KS revealed that V1, V3 and V4 were up-regulated 12.55-fold, 7.07-fold, and 10.32-fold in KR, respectively; while V2 and V5 were down-regulated 2.05-fold and 1.75-fold in KR, respectively (*P* < 0.05) (Fig. 6c). Similarly, The comparison of gene expression levels between SR and SS indicated that V1, V2, V3, V4 and V5 were respectively down-regulated 9.09-fold, 5.88-fold, 2.17-fold, 3.13-fold, and 1.69-fold (*P* < 0.05) in SR (Fig. 6c). On the other hand, as the crucial function of TIR domain in signal transduction^40^, the expression levels of V1, V2 and V3 were compared between different phenotype fish as well. To this end, another two samples named KH (head-kidney sample of health *C. idella* from the control group) and SH (spleen sample of health *C. idella* from the control group) were prepared. In all six samples, the expression level of V3 was the highest (Fig. 6d). The expression levels of V2 were significantly higher than that of V1 (*P* < 0.05) in KS, SR, and SH, and slightly higher than that of V1 in SS and KH (*P* > 0.05) (Fig. 6d). However, the expression level of V2 was lower than V1 in KR (*P* < 0.05) (Fig. 6d). From above data, it is obvious that the expression patterns of IL-1R1 between RT-qPCR and RNA-seq data in the head-kidney libraries are different. Since IL-1R1 contains various spliced transcripts, this divergence may be caused by the bias of the RNA-seq^41^. Allowing for the sequences of *IL-1R1* transcripts were Sanger-sequenced and the primers used for RT-qPCR were specific, the results from RT-qPCR may be more reliable. Faced with this contradiction, two arguments can support the reliability of RT-qPCR results. Firstly, according to the literature review^40^, the proteins ‘encoded’ by IL-1R1 V1 and IL-1R1 V3 without the TIR or/and TM modules are the negative regulation receptors in IL-1R1 signal pathway (Fig. 6b). In the latter stage of immune responses, immune system ought to inhibit the activity of inflammatory cytokines through mRNA degradation mechanism and corresponding inhibitors, making the organism return to a state of homeostasis. Due to the proteins ‘encoded’ by IL-1R1 V1 and IL-1R1 V3 are decoy receptors to sequester IL-1 signals, the relatively high expression of IL-1R1 V1 and IL-1R1 V3 and the relatively low expression of IL-1R1 V2 in KR library presented by the RT-qPCR analysis might indicate that the IL-1 signal pathway was being repressed in the head-kidney of those resistant fish. Meanwhile, all the *IL-1R1* mRNA was down-regulated, which suggested the inhibition process of IL-1 signal pathway had been completed in the spleen of those resistant fish. Secondly, the proteins ‘encoded’ by IL-1R1 V4 possess signal peptides which lead the IL-1R1 peptides to get through the cell membrane. The extracellular IL-1R1 for instance the soluble form of IL-1R1 (sIL-1R1) can capture IL-1 to prevent IL-1 from interacting with membrane receptors^40^. Thus, the relatively high expression of IL-1R1 V4 in head-kidney of resistant fish might also aim at suppressing the IL-1 signal pathway to control inflammatory response. On the other hand, the RNA-seq revealed the expression level of IL-1R1 V2 was higher than that of IL-1R1 V1 and IL-1R1 V3, but the RT-qPCR showed IL-1R1 V3 was of the highest expressed one (Fig. 6d). Inquiringly, RT-qPCR analysis of health fish also indicated IL-1R1 V3 was higher expressed than IL-1R1 V1 and IL-1R1 V2. Given the proteins ‘encoded’ by IL-1R1 V3 include sIL-1R1 and intracellular form of sIL-1R1 (icsIL-1R1)^40^, it can be concluded that sIL-1R1 and icsIL-1R1 play a pivotal role in regulating IL-1R1 signal pathway in both virus infected and healthy *C. idella*, and maybe they are involved in other physiological processes of *C. idella*. The inquiry of the rational explanation for the divergence between RT-qPCR and RNA-seq may turn to more methods, such as microarray and IVT-seq^41^. It should be noted that the expression level of IL-1R1 V2 is lower than that of IL-1R1 V1 in KR library, which may result from the degradation caused by some microRNAs for instance miR-135b^42^. Although many questions remain to answer in the present study, it is certain that IL-1R1 of *C. idella* contains 12 splicing transcripts which participate in the different regulations of IL-1R1 signal pathway.

In terms of *ILLRs* genes, because of sequences similarity between different splicing transcripts and between different genes, RT-qPCR validation of *ILLRs* was extremely difficult. As a result, there was no further verification for the expression of *ILLRs* genes. According to RNA-seq data, it was exceptional that the expression pattern of *ILLR2e* was dissimilar to that of other splice transcripts of *ILLR2*, and to be more specific, the expression levels of *ILLR2e* were lower in KR and SR than those in KS and SS, respectively (Fig. 7b). Since each gene possesses a dominant transcript^43^, the dominant transcripts of *ILLR2*, *ILLR3* and *ILLR4* should be *ILLR3a*, *ILLR3f* and *ILLR4b*, respectively in *C. idella*, judged by the expression level of each splicing transcripts. Interestingly, almost all the *ILLR* transcripts were more highly expressed in resistant libraries than in susceptible libraries except *ILLR2e*, which may suggest that protein encoded by *ILLR2e* functions differently in immune responses. To this end, the structures of the *ILLR2a* and *ILLR2e* encoded proteins were predicted (Fig. 7c). The difference between the two proteins is the length of coil-coiled region involved in crucial interaction for instance transcriptional control^44^, and this region maybe the key that leads to the unusual function of ILLR2e. Since few studies focus on *ILLR* genes, the immune mechanism is still unknown, and there is a long way to investigate the functions of ILLRs. Fortunately, the present research may reveal that *ILLR* genes are alternative spliced during GCRV infection in *C. idella*.

### Splicing factor gene expression signatures in the head-kidney and the spleen

To investigate the expression levels of splicing factors (SFs) during GCRV infection, a comprehensive list that contains 378 genes which have been previously reported to implicate in splicing was established^5^ (Supplementary Data 6), followed by the comparison of the corresponding expression profiles from RNA-seq dataset and the identification of gene expression signature (Supplementary Fig. S7). The SFs were classified into 10 categories: Zinc finger protein (ZF), DNA binding protein (DBP), helicase, heterogeneous nuclear ribonucleoprotein (hnRNP), kinase, pre-RNA processing factor (PRPF), ribosomal protein (RP), spliceosome-associated factor (SAF), small nuclear ribonucleoprotein (snRNP), and splicing-related protein (SRP). Among these categories, SRP category contained the largest number of unigenes (245 unigenes, 65%), while DBP category contained the smallest number (4 unigenes, 1%). The mean expression level of RPs in the four libraries was the highest. But some kinases such as receptor for activated C kinase 1 (RACK1), SRPs such as tubulin, ZFs like ZC3H13 were relatively highly expressed in the four libraries as well. These highly expressed SFs may play a pivotal role in the immune response of *C. idella*. Moreover, there was a little difference between the spleen and the head-kidney. For example, most mRNAs of SFs were relatively highly expressed in the KR library, but relatively lowly expressed in the SR library (Supplementary Fig. S7). Another, HSPA5, SLC7A9 and SYF1 in SRP category and RPS15A in PR category were scarcely expressed in head-kidney, while DNAJC6 in SRP category was barely expressed in spleen. These findings may illustrate that the expression pattern of splicing factors in the head-kidney is different from that in the spleen. Given these, further researches on the relationship between SFs in fish head-kidney and antiviral responses should be examined.

In summary, a relatively complete transcriptome dataset of *C. idella* was obtained, in which, many significant and novel genes can be further studied. The divergence of immune response between the head-kidney and the spleen provided by the present study may lay a foundation for the development and evolution researches of immune systems. Unlike traditional AS event analysis focusing on the adaptive immunity-related molecules such as TCR, MHC and Ig, the present study provides novel evidence for the notion that AS plays a pivotal regulatory role in immune response^1^,^6^. Since the IL-12 and IL-1R1 function in both innate and adaptive immune systems, a new question arises, that is whether some genes switch their roles in innate and adaptive immune systems via AS events? If this assumption is verified, the boundary between innate and adaptive immunity would be more unobvious^45^.

## Methods

### Ethics statement

This study was carried out in strict accordance with the recommendations in the Guide for the Care and Use of Laboratory Animals of the National Institutes of Health. The use of experimental fish was approved by the Animal Ethics Committee of Huazhong Agricultural University. In the current study, the use of tissue material from fish is a part of routine fishery production. The challenge experiment did not involve endangered or protected species. All surgery was performed under 3-Aminobenzoic acid ethyl ester methanesulfonate (MS-222) anesthesia, and every effort was made to minimize suffering.

### Fish and GCRV challenge

Healthy *C. idella* for the present experiment (approximately 10 cm in body length) were the kind gifts from Professor Hong Ji, Northwest A&F University, Shaanxi, China. Initially, fish were temporarily reared in five tanks (50 fish per tank) filled with aerated freshwater at 28 °C for adaptation. After seven days of acclimation, fish in four tanks were inoculated by intra-peritoneal injection with GCRV (097 strain, suspended in PBS) at a dose of 3.63 × 10^6^ TCID_50_/g which was applied and verified by previous challenge experiments^46^. As control, fish in the fifth tank were injected with PBS at the same dosage. After injection, all the fish were fostered under the same and suitable condition, fed with a diet according to standard feeding scheme, and continuously observed for collecting moribund animals. The head-kidney and spleen tissues of the moribund fish were instantly sampled, and then stored with RNAiso Plus (TaKaRa, Japan) at −80 °C until RNA isolation. The surviving fish after 10 days post-challenge were serenely sacrificed for head-kidney and spleen samples. RNA samples were prepared for transcriptome and gene expression analyses. Since the genetic background of these fish is unclear, we cannot know the fish is natural resistant to GCRV infection or not. Thus, to reduce the influence from genetic background and to simplify the data analysis, the RNA samples from the control group were abnegated.

### RNA isolation, library construction and sequencing

According to the result of challenge experiment, head-kidney and spleen tissues from 12 moribund fish with obvious symptom of hemorrhagic disease (muscular hemorrhage; hyperemia in operculum, fin ray, intestine and air bladder) and 12 survival fish would be used as the materials for RNA isolation, which followed the instruction of the product manual of RNAiso Plus. Total RNA content of each sample was measured by using NanoDrop 2000c UV-Vis Spectrophotometer (Thermo Fisher Scientific Inc.), and the quality of RNA samples was assessed by agarose gel electrophoresis and Agilent 2100 Bioanalyzer (Agilent technologies). According to the recommendation of TruSeq™ RNA sample preparation Guide, all samples displayed a 260/280 ratio greater than 2.0 and RNA integrity numbers (RIN) ≥8.0.

The 12 moribund fish were susceptible individuals, while the 12 survival fish were resistant individuals against GCRV infection. According to this allocation, the high-quality RNA samples from each head-kidney and spleen were divided into 4 groups: SS1 (spleen sample of susceptible fish), SR2 (spleen sample of resistant fish), KS3 (head-kidney sample of susceptible fish), KR4 (head-kidney sample of resistant fish). Equal amount of 4 grouped RNA samples were then respectively pooled for cDNA synthesis and RNA-seq. Before the construction of library, ribosomal and viral RNA were removed and poly(A)+ mRNA were isolated with magnetic Oligo-dT beads (Invitrogen, USA), and then cDNA libraries were constructed and sequenced by Majorbio Biotech Co., Ltd, Shanghai, China. Briefly, 8 μg of total RNA for each group was used for the construction of libraries by using Truseq^TM^ RNA sample prep Kit (Illumina, USA) according to the manufacturer’s protocol. The constructed DNA template was enriched by PCR amplification (15 cycles). Amplicons were collected and purified by Certified Low Range Ultra Agarose (Bio-Rad, USA) gel electrophoresis. Before sequencing, the DNA libraries were quantified by using TBS-380 micro fluorometer with Picogreen^®^ reagent (Invitrogen, USA). Clone clusters were generated on Illumina cBot, using Truseq PE Cluster Kit v3-cBot-HS, and high-throughput sequencing was performed on an Illumina Miseq sequencer, using Truseq SBS Kit v3-HS 200 cycles.

### *De novo* assembly and sequence analysis

The raw paired end (PE) reads were cleaned by removing adapter sequences, empty reads, and low quality sequences (reads with over 10% unknown base pairs ‘N’). The reads obtained were randomly decomposed into overlapping k-mers (default k = 25) for assembly by using Trinity software (http://trinityrnaseq.sourceforge.net/, version: trinityrnaseq-r2013-02-25)^47^. After assembly, the open reading frame (ORF) of each unisequence was predicted by using Trinity software. Unisequences were used for BLAST search and annotation against the NR, NT, STRING (http://string-db.org/)^48^, COG (http://www.ncbi.nlm.nih.gov/COG/), KEGG (http://www.genome.jp/kegg/) databases using an E-value cut-off of 10^−5^ 49. Functional annotation by gene ontology (GO, http://www.geneontology.org) terms was analysed by using BLAST2GO software (NCBI)^50^.

The read counts were further normalized into FPKM values. The FPKM values from the four libraries were pairwise compared, and the fold changes were calculated by using RSEM software (version 1.2.7)^51^, and DEGs were identified by using edgeR software package (version 3.8.2)^52^ (if *P* < 0.05 and FDR < 0.05, then the result would be considered statistically significant). Subsequently, the enrichment analysis of GO and KEGG pathways was performed based on these DEGs by using Goatools software (version 0.4.7) (https://github.com/tanghaibao/Goatools) and KOBAS 2.0 software (http://kobas.cbi.pku.edu.cn/home.do)^53^. All the heat maps for gene clustering in the present study were depicted by using R program (http://www.R-project.org/).

### Gene expression validation by RT-qPCR

To validate RNA-seq results in terms of DEGs, RT-qPCR was performed by using a CFX96 Multicolor Real-time PCR Detection System (Bio-Rad). To this end, another RNA samples collected from the challenge experiment was reverse transcribed by using M-MLV reverse transcriptase (Promega, USA) and random hexamers primer. To be more specific, 10 μL (approximately 5 μg) RNA was incubated with 2 μL of 50 μM random hexamers primer for 5 min at 70 °C. After incubation, 4 μL of 10 × cDNA synthesis buffer, 2 μL of 10 mM dNTPs, 20 U of RiboLock™ RNase Inhibitor (MBI), 200 U of M-MLV reverse transcriptase, and 0.5 μL of H_2_O (RNase-free) were added, and the mixture was incubated for one hour at 42 °C. Subsequently, the concentration of newly synthesized cDNA was diluted to 200 ng/μL before storing at −20 °C until it was used for further analysis. Sequence specific primers were designed by using Premier Primer 5 software (Supplementary Data 7). Among the DEGs, 36 genes were chosen for their drastically differential expressions or for their involvement in immunity. In the test, *18S rRNA* and *EF1α* served as reference genes^54^. In addition, the expression of *RAG1* and *RAG2,* two key members of adaptive immune system, whose products initiate the V(D)J rearrangements^55^, were validated as well. Finally, RT-qPCR and its data analysis were performed according to the protocol and method as previously described^54^.

### AS screening and validation

After unigenes with multiple transcripts were sorted, AS screening was performed to further select unigenes whose different transcripts were differentially expressed between 4 libraries based on the differential expression analysis. Among the target unigenes, several immune-related genes were noted. Subsequently, 5′-, 3′-terminal and genomic DNA regions of these genes were amplified and sequenced for AS event validation. Furthermore, the expression levels of these splicing transcripts were validated by RT-qPCR (*18S rRNA* and *EF1α* served as internal control). Primers for RT-qPCR were designed at the divergence regions from the splice variants of each corresponding gene by using Premier Primer 5 software, which were showed in Supplementary Data 7.

## Additional Information

**How to cite this article**: Wan, Q. and Su, J. Transcriptome analysis provides insights into the regulatory function of alternative splicing in antiviral immunity in grass carp (*Ctenopharyngodon idella*). *Sci. Rep.* **5**, 12946; doi: 10.1038/srep12946 (2015).

## Supplementary Material

Supplementary Information

Supplementary Dataset 1

Supplementary Dataset 2

Supplementary Dataset 3

Supplementary Dataset 4

Supplementary Dataset 5

Supplementary Dataset 6

Supplementary Dataset 7

## Figures and Tables

**Table 1:** Summary of the transcriptome of *C. idella*.

	SS1	SR2	KS3	KR4
MiSeq statistics				
Raw reads	32859452	19492284	24450204	35183620
Average read length (bp)	216	216	214	217
Total base pairs (bp)	7095838055	4215899019	5219216274	7631346510
≥Q20^a^ of clean reads (%)	94.91	90.15	91.74	94.56
Clean reads	32535864	17678754	22841432	34903598
Average read length (bp)	204	191	193	204
Total base pairs (bp)	6627953846	3374188672	4406813074	7112896707
≥Q20 of clean reads (%)	96.34	92.31	93.82	95.83
Assembly statistics (all the clean reads from the 4 libraries were together assembled)				
Total unisequence	101812			
Total unigene	55199			
Total residues (bp)	149640982			
Average length (bp)	1470			
N50^b^ unigene size (bp)	2350			
Length of the largest unisequence (bp)	18562			
Length of the smallest unisequence (bp)	351			

^a^Q20: percentage is the proportion of nucleotides with a quality value >20 in reads.

^b^N50: unigene length-weighted median.
