# Supplementary material for: Transcriptome analysis provides insights into the regulatory function of alternative splicing in antiviral immunity in grass carp (Ctenopharyngodon idella)
Source: Sci Rep. 2015 Aug 7;5:12946. doi: 10.1038/srep12946 (PMC4528194; doi:10.1038/srep12946)
Supplement: Supplementary Information [file srep12946-s1.pdf]

Transcriptome analysis provides insights into the regulatory function of alternative splicing in antiviral immunity in grass carp (*Ctenopharyngodon idella*)

Quanyuan Wan[1,2] & Jianguo Su[1,2]

[1]College of Fisheries, Huazhong Agricultural University, Wuhan 430070, China

[2]Freshwater Aquaculture Collaborative Innovation Center of Hubei Province, Wuhan 430070, China

Correspondence and requests for materials should be addressed to J. S. (email: sujianguo@mail.hzau.edu.cn)

Supplementary Figures

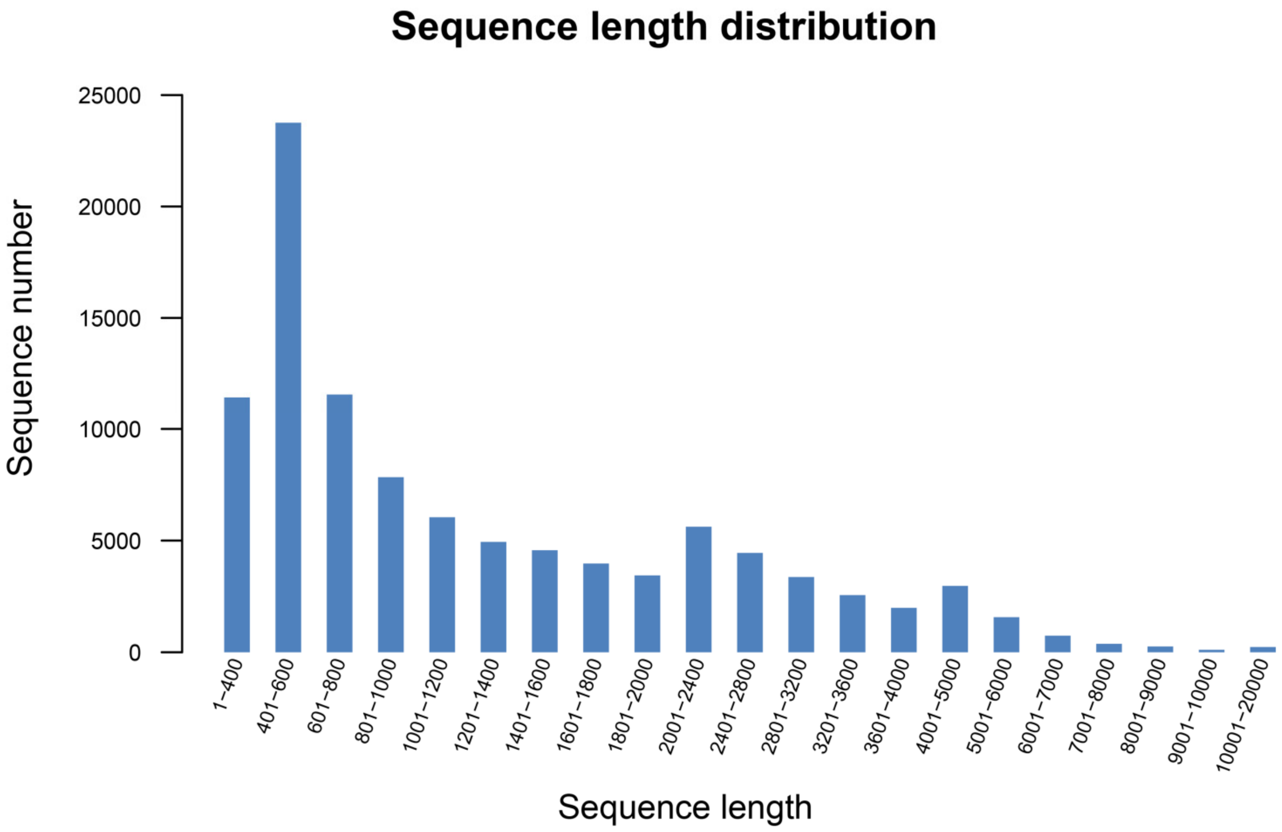

Supplementary Figure S1 | Distribution of lengths of unisequences from the finally assembled dataset.

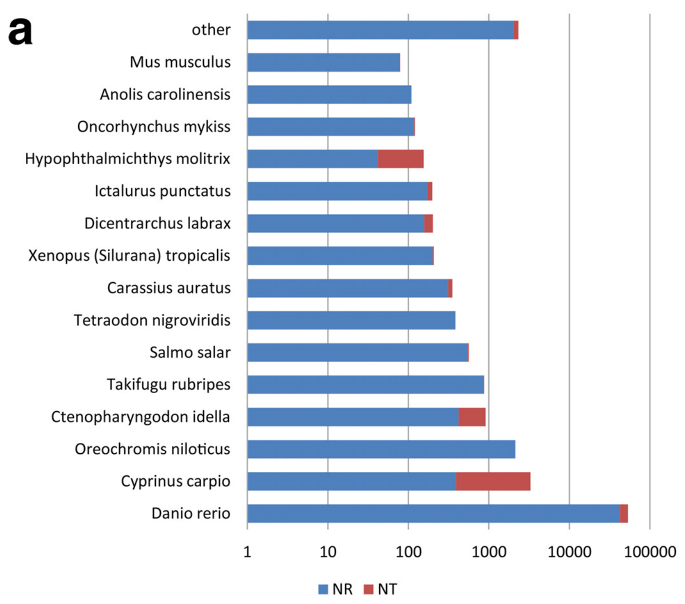

Supplementary Figure S2 | BLAST top-hit species distribution of gene annotation showing high homology to NT and NR databases. Abscissa presented the BLAST hits. a) BLAST hits that were mainly mapped to fish species; b) BLAST hits that were mainly mapped to non-fish species. Only 0.9% of the BLAST hits matched C. idella protein and nucleotide sequences due to the extremely limited number of the *C. idella* sequences that are currently available in the NCBI database.

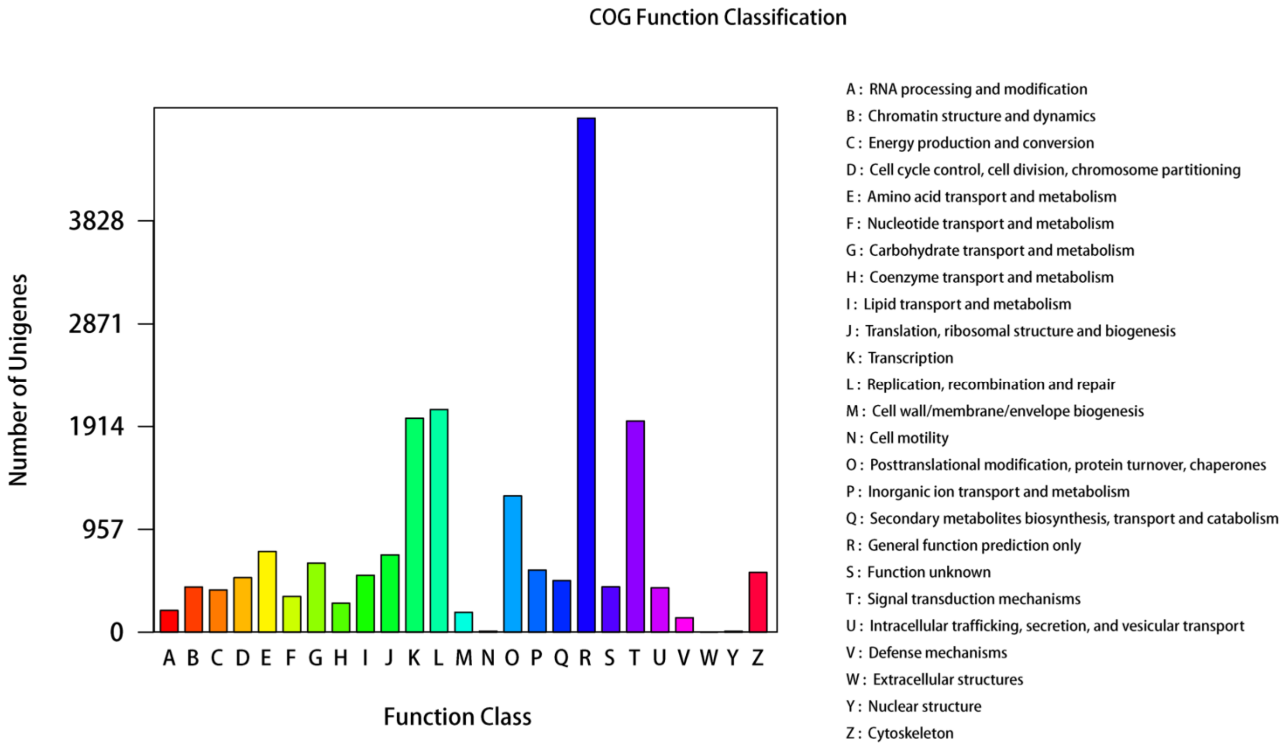

Supplementary Figure S3 | COG annotations of putative proteins. All putative proteins can be classified functionally into at least 25 molecular families in COG database.

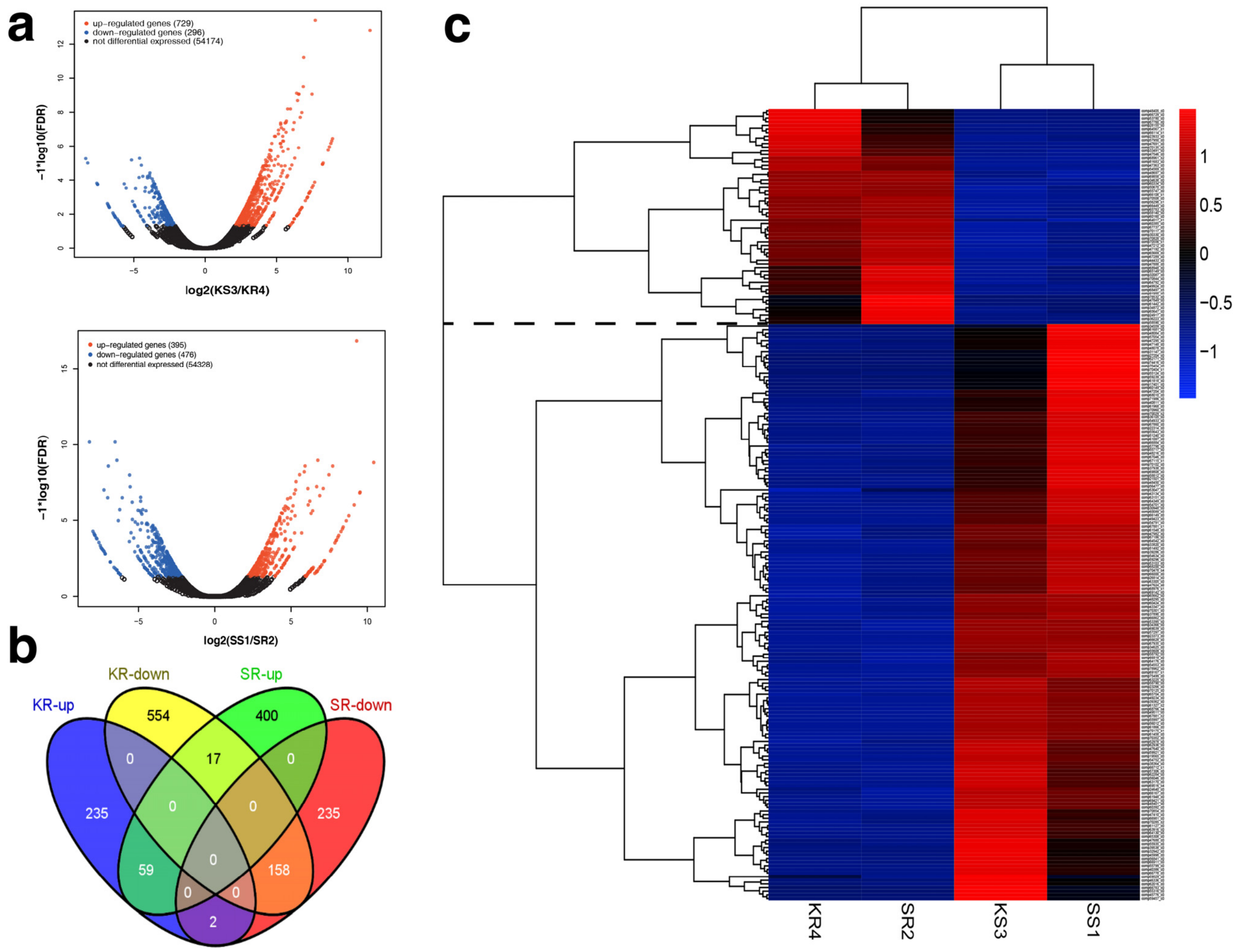

Supplementary Figure S4 | DEGs between susceptible and resistant libraries. a) Volcano plots from the differential expression analysis of the transcriptomes of head-kidney (KS3/KR4) and spleen (SS1/SR2). The $-\log_{10}$ of FDR is used for the Y axis and the $\log_2$ of the fold change is used for the X axis. b) Venn diagrams of above data, illustrating the overlap of up-regulated and down-regulated DEGs in head-kidney and spleen in the comparison of resistant and susceptible libraries. c) Two-dimensional hierarchical clustering result. It was performed based on the FPKM values of DEGs which were significantly up- or down-regulated in both head-kidney and spleen. In the comparison between resistant libraries and susceptible libraries, 59 up-regulated genes (over the dotted line) and 158 down-regulated genes (below the dotted line) in resistant libraries were found.

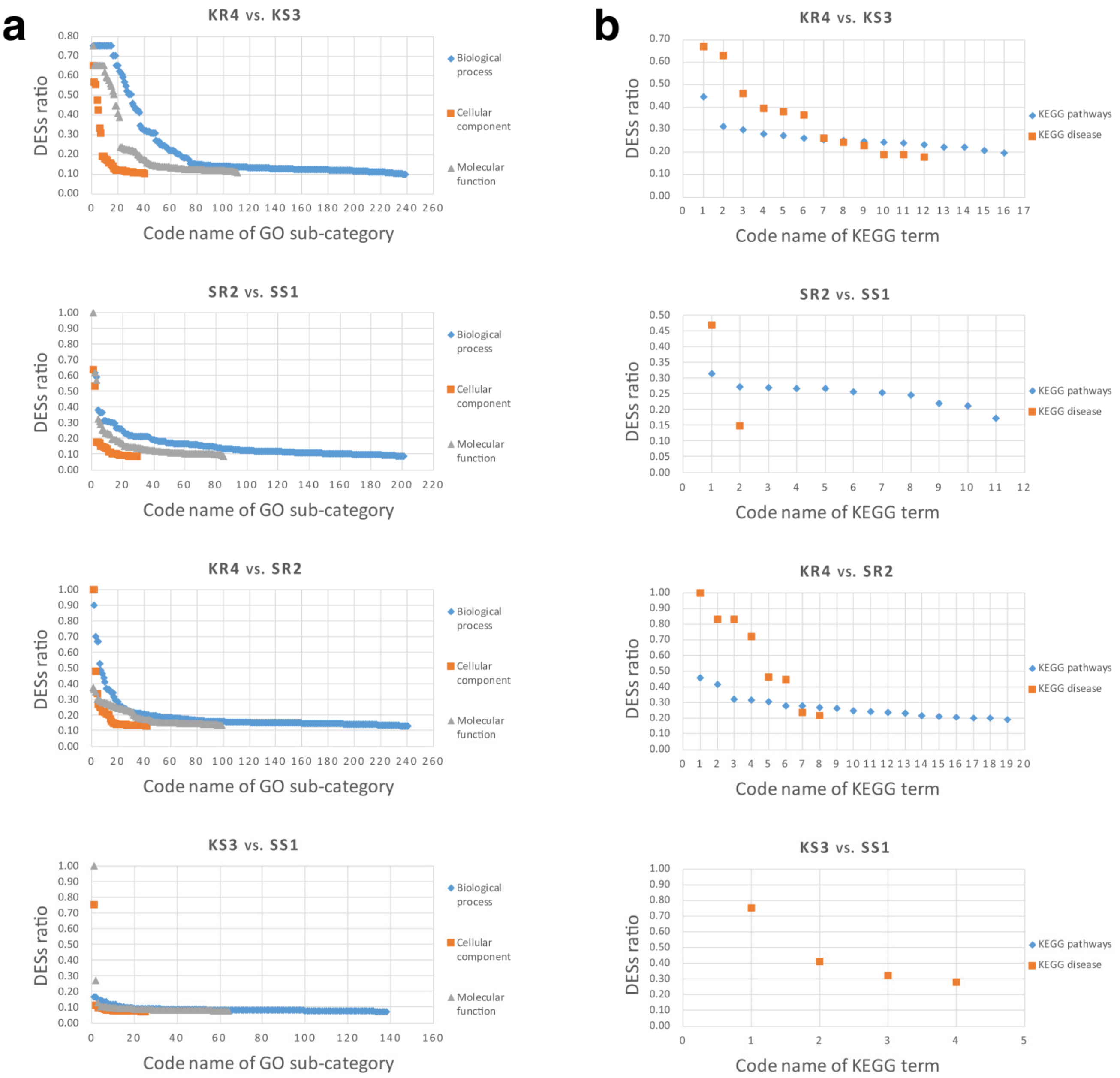

Supplementary Figure S5 | Enrichment analyses for DESes. DESes were classified into various GO categories and KEGG terms ($P < 0.05$, $FDR < 0.05$). X axis presents the code names of corresponding terms (the actual names are described in Supplementary information S6); Y axis presents DES ratios. a) GO enrichment. b) KEGG enrichment. In the comparison of KR4 vs. KS3, GO sub-categories with the highest enrichment DES ratios in BP, CC and MF category are "regulation of antigen processing and presentation", "TAP complex" and "protein N-acetylglucosaminyltransferase activity", respectively; KEGG terms with the highest enrichment DES ratios were "collecting duct acid secretion" pathway and "hepatic porphyria" disease. In the comparison of SR2 vs. SS1, GO sub-categories with the highest enrichment DES ratios in BP, CC and MF category are "toxin metabolic process", "hemoglobin complex" and "uridine phosphorylase activity", respectively; KEGG terms with the highest enrichment DES ratios were "methane metabolism" pathway and "thalassemia" disease. In the comparison of KR4

vs. SR2, GO sub-categories with the highest enrichment DES ratios in BP, CC and MF category are “spindle midzone assembly involved in mitosis”, “condensin complex and central spindlin complex” and “serine-type endopeptidase inhibitor activity”, respectively; KEGG terms with the highest enrichment DES ratios were “renin-angiotensin system” pathway and “afibrinogenemia” disease. In the comparison of KS3 vs. SS1, GO sub-categories with the highest enrichment DES ratios in BP, CC and MF category are “regulation of RNA splicing”, “ER to Golgi transport vesicle” and “glucagon receptor binding”, respectively; KEGG terms with the highest enrichment DES ratios is “skin fragility-woolly hair syndrome” disease (no significant pathway enriched).

a

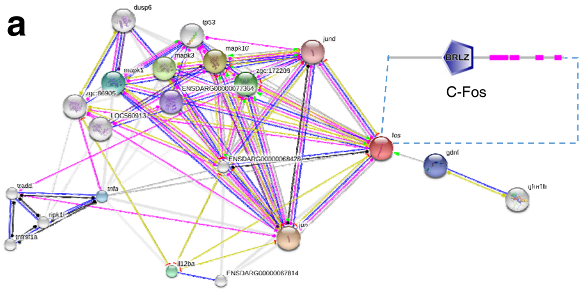

Predicted Functional Partners		Activation	Inhibition	Banding	Phnotype	Catalysis	Post-transl.m	Reaction	Expression	Score
jun	c-jun protein	◦	●				◦		◦	0.997
mapk10	Mitogen-activated protein kinase 10	◦					◦		◦	0.974
mapk3	Mitogen-activated protein kinase 3		◦				◦		◦	0.951
zgc: 172209	Mitogen-activated protein kinase 8	◦					◦		◦	0.949
Il12ba	Interleukin 12B								◦	0.938
Mapk1	Mitogen-activated protein kinase 1		◦				◦		◦	0.937
tnfa	Tumor necrosis factor α	◦								0.933
gdnf	Glial cell line-derived neurotrophic factor	●								0.928
ENSDARG00000077364	mitogen-activated protein kinase 9 isoform JNK2 alpha2-like									0.924
jund	jun D proto-oncogene	◦	●				◦		◦	0.923

b

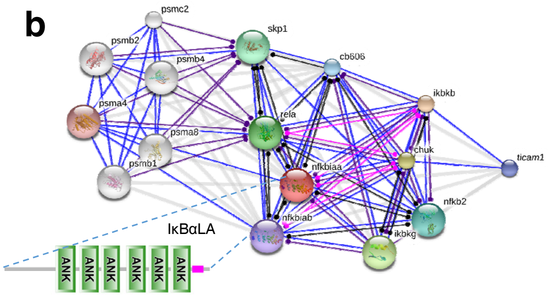

Predicted Functional Partners		Activation	Inhibition	Banding	Phnotype	Catalysis	Post-transl.m	Reaction	Expression	Score
Ikbkb	inhibitor of nuclear factor kappa B kinase beta subunit			●		●	◦			0.997
chuk	Inhibitor of nuclear factor kappa-B kinase subunit alpha			●		●	◦			0.974
ikbkg	inhibitor of kappa light polypeptide gene enhancer in B-cells, kinase gamma			●		●				0.951
rela	v-rel reticuloendotheliosis viral oncogene homolog A			●					●	0.949
skp1	S-phase kinase-associated protein 1			●					●	0.938
nfkb2	nuclear factor of kappa light polypeptide gene enhancer in B-cells 2			●					●	0.937
cb606	si-dkeyp-110c7.1								●	0.933
ticam1	toll-like receptor adaptor molecule 1									0.928
nfkbiab	nuclear factor of kappa light polypeptide gene enhancer in B-cells inhibitor, alpha b			●					●	0.924
psma4	proteasome alpha 4 subunit			◦						0.923

Supplementary Figure S6 | Predicted protein-protein interaction networks. a) The interaction network of c-Fos. c-Fos represented by the red boll was predicted to be activated by glial cell line-derived neurotrophic factor (GDNF) which interacted with GDNF family receptor alpha 1b (GFRa1b). The detail cooperation information is showed in the right chart. Beside the network graph is the predicted protein structure of c-Fos. b) The interaction network of IκBαLA (the red boll). It is much complex that IκBαLA can interact with IKBKB (inhibitor of nuclear factor kappa B kinase beta subunit), IkBKA, IKBKγ, RELA (v-rel reticuloendotheliosis viral oncogene homolog A), proteasome subunits, etc. And the detail information and the predicted structure of IκBαLA are displayed as well.

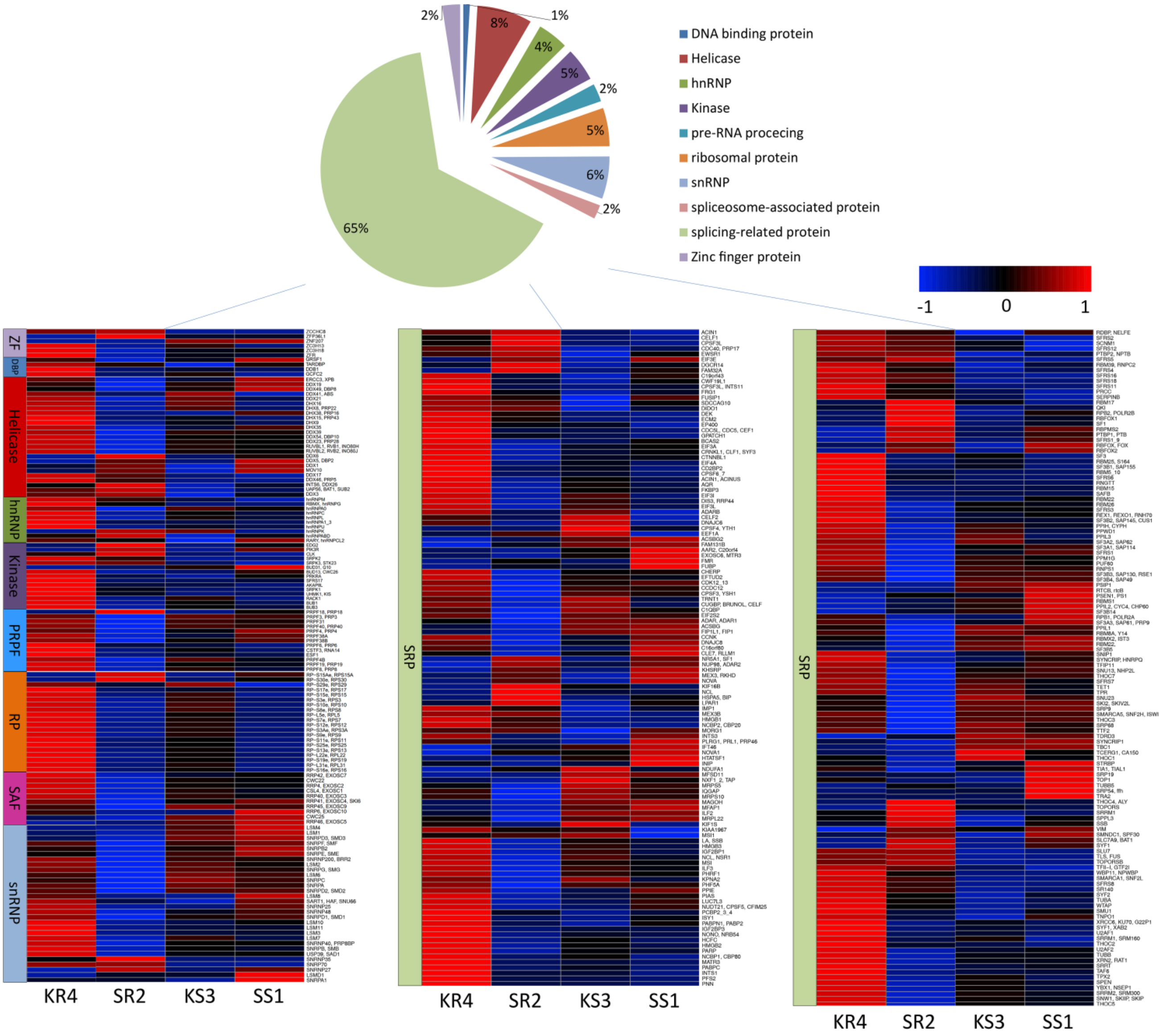

Supplementary Figure S7 | Various SFs in the four libraries of *C. idella* during GCRV infection. Pie graph shows the ratios of these ten classifications of SFs. Heat map depicts the variation in expression of SFs. The average FPKM value of each gene is the evaluation criteria, that is, genes with expression levels greater than the mean are coloured red and those below the mean are coloured blue.

Supplementary Tables

Supplementary Table S1 | The mapping result of high-quality with the assembled transcriptome.

	SS1	SR2	KS3	KR4
**Mapped reads**	27,172,728	14,806,340	19,098,334	29,956,628
**Percentage of mapped reads**	83.52%	83.75%	83.61%	85.83%

Supplementary Table S2 | Data summary of the functional annotation of *de novo* assembled sequences of *C. idella*.

	NT	NR	COG/KOG/NOG	GO	KEGG	Total annotated
Unisequence	67,201	55,700	34,071	32,519	27,367	73,828
DES	30,561	21,473	14,337	14,188	11,602	34,567

Note: DES is the abbreviation of differentially expressed unisequence.

Supplementary Table S3 | RT-qPCR confirmation of the 38 significantly differentially expressed genes.

Gene ID	Annotation	RNA-Seq		RT-qPCR (ref. *EF1α*)		RT-qPCR (ref. *18S*)	
		KR vs. KS	SR vs. SS	KR vs. KS	SR vs. SS	KR vs. KS	SR vs. SS
**comp36223_c0**	TNNC2	6.76	7.84	4.18	1.28	4.57	0.86
**comp47888_c0**	New	6.02	6.19	4.10	0.02	4.17	-0.41
**comp49924_c0**	ADP/ATP translocase	4.28	7.50	3.60	1.19	3.58	0.85
**comp28155_c0**	New	7.54	4.42	4.35	1.61	4.42	1.27
**comp48697_c0**	TLR19	3.11	7.35	3.26	1.02	3.53	0.69
**comp91950_c0**	M3-CK	6.35	5.06	4.33	0.91	4.48	0.49
**comp24917_c0**	New	4.94	5.40	3.30	-0.75	3.44	-1.33
**comp66108_c1**	New	5.09	3.81	4.63	4.97	4.82	4.79
**comp78532_c0**	parvalbumin	2.89	5.03	4.63	0.87	4.77	0.35
**comp68961_c2**	New	3.66	4.12	3.74	0.88	3.96	0.36
**comp67760_c0**	CAND1	4.37	3.39	3.32	0.43	3.49	0.14
**comp34872_c0**	novel alpha globin	3.55	3.48	2.83	6.17	3.19	6.11
**comp30330_c0**	New	4.51	3.13	3.83	0.72	3.90	0.25
**comp44433_c0**	c-Fos	2.60	3.17	2.83	2.50	3.01	2.06
**comp47192_c0**	alpha globin	3.54	2.85	4.65	5.17	4.73	4.87
**comp70556_c1**	beta globin	3.45	2.69	3.95	5.03	4.07	4.74
**comp68948_c0**	LLRC15-like	3.06	2.81	4.99	4.44	5.12	4.14
**comp61896_c0**	RAG1	2.00	-2.71	3.60	-2.78	3.72	-3.19
**comp60942_c0**	RAG2	1.22	-0.09	2.90	-1.47	2.90	-1.47
**comp59457_c0**	New	-6.54	-6.41	0.17	-3.35	0.41	-3.71
**comp67935_c0**	NMB	-6.66	-6.23	-2.99	-6.65	-2.84	-6.96
**comp61818_c0**	MART1-like	-6.07	-6.88	-0.92	-3.30	-0.80	-3.71
**comp62284_c0**	New	-5.59	-7.59	-1.39	-2.48	-1.32	-3.01
**comp61097_c0**	CIPK26	-4.37	-9.14	-0.43	-3.99	-0.28	-4.46
**comp49423_c0**	New	-6.71	-7.02	-0.68	-5.22	-0.46	-5.59
**comp23266_c0**	New	-7.17	-6.64	-3.50	-8.70	-3.38	-9.21
**comp60089_c0**	BMP2-like	-5.71	-8.37	-1.06	-3.72	-0.88	-4.27
**comp56046_c0**	inhibin	-6.23	-9.53	-0.92	-4.15	-0.69	-4.77
**comp46336_c0**	New	-8.80	-6.92	-0.92	-2.07	-0.71	-2.34
**comp23373_c0**	proteasome subunit	-8.27	-7.95	-4.15	-6.22	-4.03	-6.69
**comp37890_c0**	NF-κB1 enhencer	-2.31	-2.25	-2.32	-1.15	-2.20	-1.57
**comp66783_c0**	IL-1β	-4.00	-3.15	-0.37	-2.44	-0.24	-2.90
**comp58812_c0**	IFNγR1	-3.38	-4.09	1.71	-4.49	1.81	-5.02
**comp67115_c1**	IL-11	-4.62	-3.86	-2.41	-5.71	-2.28	-6.13
**comp34625_c0**	IL-8	-3.90	-4.62	-5.92	-4.39	-5.96	-4.68
**comp34559_c0**	CCL-C24c	-4.12	-5.92	-0.45	-5.62	-0.38	-6.08
**comp48078_c0**	IFNγ2a	-5.69	-5.87	1.18	-4.98	1.30	-5.38
**comp66712_c0**	iNOS	-7.71	-9.30	-9.37	-10.87	-9.36	-11.28

Note: the numbers are the $\log_2$(fold change) of corresponding gene. "New" presents there is no annotated information of corresponding gene in the NR database.
